# The effect of B-type allatostatin neuropeptides on crosstalk between the insect immune response and cold tolerance

**DOI:** 10.1038/s41598-022-25235-w

**Published:** 2022-11-30

**Authors:** Jan Lubawy, Justyna Hornik

**Affiliations:** Department of Animal Physiology and Developmental Biology, Institute of Experimental Biology, Faculty of Biology, Adam Mickiewicz University, Poznan, Poland

**Keywords:** Neuroimmunology, Neurophysiology, Peptides

## Abstract

Insects are the largest group of arthropod phyla and are capable of surviving in a variety of environments. One of the most important factors in enabling them to do so is their resistance to temperature stress, i.e., cold tolerance. The neuroendocrine system, together with the immune system, cooperates to regulate a number of physiological processes that are essential for the stability of the organism in stressful conditions. However, to date, no one has studied the effect of insect myoinhibitory peptides (MIPs) on cold stress tolerance and immune system activity. Here, we investigated the effect of Tenmo-MIP 5 (10^–6^ M), cold stress (− 5 °C) and a combination of both on the immune response of *Tenebrio molitor*. All three treatments caused upregulation of immune-related genes (antimicrobial peptides and Toll) and increased phagocytosis activity (by approximately 10%). However, phenoloxidase activity and mortality were increased only after peptide injection and the combination of both treatments. The peptide injection combined with cold stress caused 40% higher mortality than that in the control. Together, our results show the links between cold stress, MIPs activity and the immune response, and to our knowledge, this is the first report showing the effect of MIP on the insect immune system.

## Introduction

Within arthropods, insects are the most numerous representatives. They are able to survive in almost every environment from tropical regions of Africa through meadows of temperate regions to the freezing Arctic^1^. One of the most important elements shaping the distribution of insect species is their tolerance to temperature, more precisely their cold tolerance, and water availability^2^. Over the course of their evolution, insects exposed to low temperatures evolved different adaptations to prevail and prosper in suboptimal conditions^3,4^. On the basis of their ability to survive cold/freeze stress, insects traditionally are divided into a couple of separate categories: (a) freeze-tolerant—species capable of tolerating freezing of their tissues; (b) freeze-avoidant—insects from this category survive cold stress by lowering their freezing point through a phenomenon known as supercooling; (c) chill-tolerant—these species possess a low supercooling point (SCP, between − 20 and − 30 °C) and a high level of cold tolerance, but unlike freeze-avoiding insects, some mortality above the SCP occurs in this group; (d) chill susceptible—species from this group die after short exposures (minutes or hours) at temperatures often substantially above the SCP; and (e) opportunistic survivals—species from this group are unable to survive below the threshold temperature for development and must therefore ‘opportunistically’ locate sheltered overwintering sites^5,6^. More recently, it was proposed to categorize insects due to types of injury: (I) related to ice formation, further divided into freeze-tolerant and -avoiding; and (II) related to chilling further divided into chill-tolerant and -susceptible (for details see^7–9^ and references within).

All physiological processes, including those that characterize insects belonging to the abovementioned groups, are governed by two inseparable systems, nervous and endocrine^10^. Through the process called neuroendocrine integration, the nervous and endocrine systems, together with a third system—immune system—interplay and communicate together to regulate a number of physiological processes that are essential for the stability of the organism in regular as well as stressful situations^11–13^.

One of the most important types of molecules in the nervous system of all living animals are neuropeptides. They play a fundamental role in the control of physiological processes and constitute one of the most diverse groups of signalling molecules in terms of function^14–16^. They can act as neurohormones, neuromodulators and neurotransmitters^17,18^. They are produced mainly in the central nervous system (CNS) and take part in the regulation of metabolism, ion homeostasis and muscle contractions, including the heartbeat^19^. In different insect species, neuropeptides with homologous structures very often have similar functions and have been identified as signalling molecules mediating communication between the three systems^20^. Because neuropeptides play central roles in physiological processes, directly affecting the survival of adverse conditions, molecules such as, e.g., capability peptides (CAPA), inotocin (ITC) or diuretic hormones (DH_31_ and DH_44_), which are responsible for osmoregulation, will take part in the response to cold stress. Additionally, compounds related to the general stress response, such as tachykinin-related peptides (TRPs) and/or peptides responsible for the regulation of carbohydrates and lipoproteins, such as adipokinetic hormones (AKHs), insulin-like peptides (ILPs) and juvenile hormone (JH), take part in the response to cold stress^10^.

Stress-response signalling pathways have been well explored and are highly interactive. Such interplay between regulatory pathways is often referred to as “cross-talk” and is defined as shared regulatory or signalling pathways that initiate distinct protective mechanisms against different stressors^21^. Insects equipped with innate immunity have mechanisms of cellular and humoral response, allowing them to fight off invading pathogens such as bacteria, fungi, viruses and parasites^22–24^. Additionally, it has been established that some cellular protection mechanisms are efficient against different forms of stress; for example, organic molecules such as amino acids, polyols and sugars can protect cells against thermal, osmotic, and several other stresses^25^. These are good examples of “cross-tolerance”—mechanisms that guard the organism from two or more types of stress^21^.

Among insect neuropeptides, one of the largest groups is allatostatins (ASTs). Although ASTs are grouped into one large family due to their activity on the synthesis of juvenile hormone (JH), many studies have shown that this might not be their only role in the insect body^26,27^. ASTs show pleiotropic activity affecting vitellogenesis^28,29^, synthesis of digestive enzymes^30^, and visceral muscle contractions^31^. Allatostatins are also considered a homologue of vertebrate somatostatin, and receptor homology also shows that they are descended from the same origin^17^. In vertebrates, somatostatin and substance P are directly involved in immune system regulation^13,32^.

Due to all of the above, we decided to test the effect of Tenmo-MIP 5 on the activity of the immune system, cold stress tolerance and the crosstalk between the two in *Tenebrio molitor* L beetle. To achieve this, we measured the expression of genes encoding antimicrobial peptides and the Toll pathway, phenoloxidase activity, phagocytosis and total haemocyte count after peptide injection, cold stress or a combination of both. We also measured the time to recover from chill coma after cold stress together with peptide injection and conducted survival experiments showing that the combination of neuropeptide injection and cold stress lowers the cold tolerance of beetles.

## Materials and methods

### Insect rearing and tissue collection

**Figure 1:**
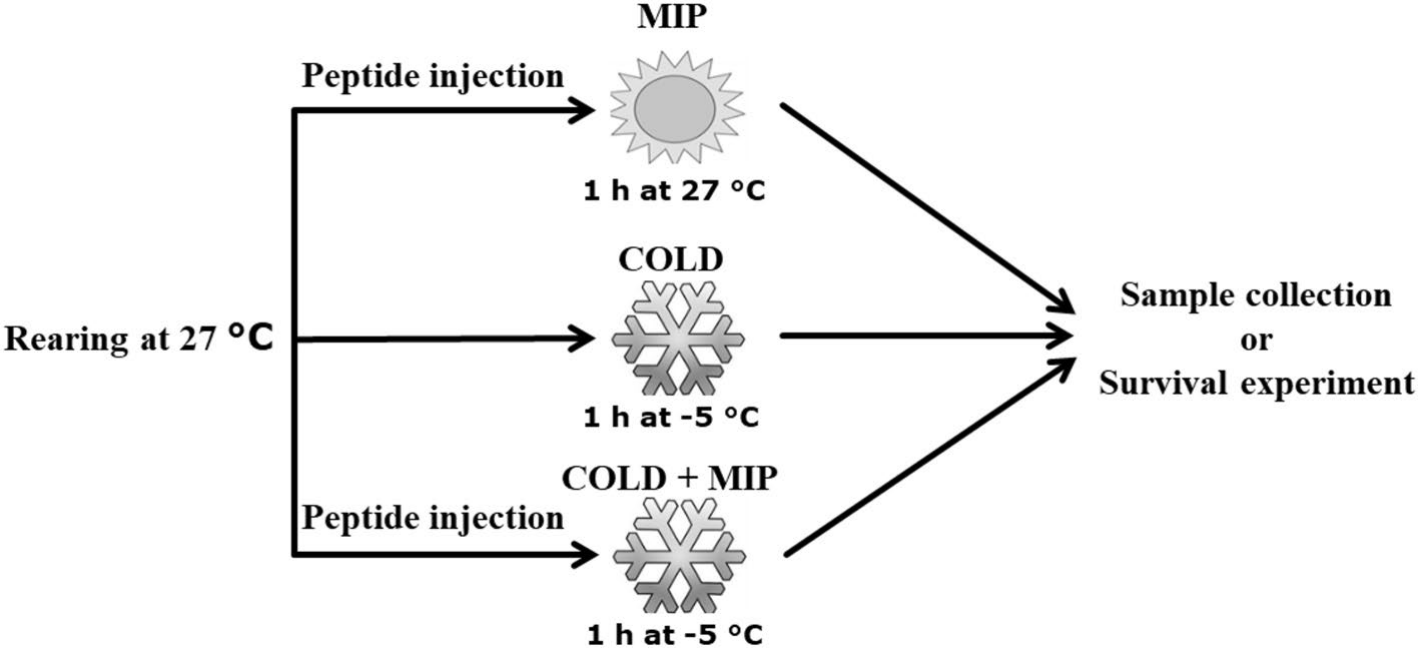
Scheme of the experimental designs.

One-month-old adults of *T. molitor* were obtained from a colony maintained in the Department of Animal Physiology and Developmental Biology at Adam Mickiewicz University, Poznań, Poland. The beetles were reared as previously described^33,34^. The insects were subjected to the following treatments: either injected with Tenmo-MIP 5 (denoted MIP), subjected to cold stress (denoted COLD) or injected with Tenmo-MIP 5 and subjected to cold stress (denoted COLD + MIP) (Fig. 1). For the control, we used untreated beetles or beetles injected with physiological saline (PS, 274 mM NaCl, 19 mM KCl, 9 mM CaCl_2_). Before sample collection (haemolymph, fat body or neural tissues) or neuropeptide injection, the insects were anaesthetized with CO_2_. Haemolymph samples were collected by cutting the tibia of the first pair of legs. This method allows a sample of haemolymph to be obtained without external contamination. Whole brains, ventral nerve cords (VNC) and fat body tissue (FB) of *T. molitor* were dissected in PS under sterile conditions using a Zeiss Stemi 508 stereomicroscope.

### Peptide injection

Tenmo-MIP 5 was synthesized by Creative Peptides (Shirley, New York, USA). The synthetic peptides were dissolved in PS to produce 1 mmol/L stock solutions, which were stored at − 30 °C. Working solutions were made from the stock solution in PS just before the assays were carried out. Two microlitres of neuropeptide solution was injected 1 h before experiments at a concentration of 10^–6^ M. Based on the literature data, the final concentration of neuropeptide in the insect body was 10^–7^ M (Marciniak et al. 2017; Urbański et al. 2021). Neuropeptide solution was injected under the left coxa of the third pair of legs using 5 µL Hamilton® Syringe (Model 87908 with needle style number 4—for biological sciences). To avoid accidental damage to the gut, which could cause the leakage of digestive enzymes into the body cavity, the needle was inserted parallel to the long axis of the insect's body to a depth of approximately 1.5–2 mm.

### Thermal treatments

Insects were subjected to cold stress by placing three individuals in 2 ml Eppendorf tubes, which were submerged in a cryostat bath (Ministat 120, Huber Kältemaschinenbau AG, Germany) filled with ethanol. The temperature of the bath was set to − 5 °C. Insects were left in the bath for 1 h, after which they were removed and placed in a plastic container until they recovered (coordinated movement) and then taken for further analyses.

### Chill coma recovery test

After COLD and MIP + COLD treatment, we measured the beetles' recovery time from chill coma (CCRT), a common method of determining cold tolerance in insects^35^, to establish the effect of peptide injection on the time to recover from coma and beetle cold tolerance. In the case of these procedures, insects from COLD were treated as a control. Additionally, we measured CCRT in PS treatment to account for the effect of the injection itself on the measured parameter. To measure the CCRT, after each treatment, beetles were placed individually in square plastic boxes and returned to room temperature (22 ± 0.5 °C). The recovery time was visually scored using a timer started immediately after removing beetles from the cryostat bath. The time it took beetles to regain coordinated movements of the legs was called CCRT^36^.

### Survival experiments

After each treatment (COLD, MIP or COLD + MIP), 10 individuals were placed in a breeding room in plastic boxes (15 × 30 × 20 cm) with carrots for food to test for survival after stress exposure. Mortality was recorded at 1 h after stress and each day thereafter for a period of 21 days. The insects were considered dead when they did not react to the pinch of the legs and antenna using forceps. An untreated control group and group injected with PS of 10 insects were also monitored during the same period. Each research variant was repeated at least 3 times (3 × 10 individuals = 30 beetles per treatment).

### mRNA isolation and generation of cDNA

After tissue isolation, ten samples of neural tissues (10 × brains and 10 × VNCs) and four to five of FB were pooled together for one biological repetition to extract the total RNA using the Insect RNA MicroPrep Kit (Zymo Research Corp., Irvine, CA, USA) according to the manufacturer’s protocols. The protocol included in-column DNase I treatment to remove traces of gDNA and prevent contamination of the RNA samples. ReverAid Reverse Transcriptase (Fermentas, Waltham, MA, USA) was then used to generate cDNA for PCR according to the manufacturer’s protocols. To evaluate the level of Tenmo-MIP after cold stress in the tested tissues, we used primers previously described by Lubawy et al.^37^ (Table 1), and to measure the level of immune-related genes, we used primers previously published by Jacobs et al.^38^ (Table 1) or Urbański et al.^39^. All primers were synthetized by the Institute of Biochemistry and Biophysics of the Polish Academy of Sciences in Warsaw. Quantitative reverse real-time PCR (RT‒qPCR) was performed on a Corbett Research RG-6000 Real Time PCR Thermocycler (QIAGEN, Hilden, Germany) using a Sensitive RT HSPCR Mix SYBR kit (A&A Biotechnology s.c, Poland). As the reference gene, *T. molitor* ribosomal protein L13a (TmRPL13a) was used to normalize differences in template concentration between samples. To check for potential foreign contamination of samples, “no template control” (DNA/RNA free water) and “no RT control” reactions were also included in the analysis. For each research variant and control, 3 biological repetitions in 2 technical replications were performed. All results were standardized using the *RPL13a* gene, and relative expression was calculated using the 2^-^^ΔΔ^Ct method.

**Table 1:** Sequences of primers used in the study.

Name	Forward primer	Reverse primer	References
Toll	TGCGTAGCAAACAGGTGGAT	TCGCGTAGCGGTAGTAGAGA	Jacobs et al.^38^
Attacin-A	CGAAGCAGTTCCGTCCATCT	CTCCTCCACAGGTTCGCATT	Jacobs et al.^38^
Cecropin	ATGGACAACCAATGCCACCC	GGTCTTCGATTCCGTTGCCT	Jacobs et al.^38^
Coleoptericin-A	TTGGGATTCTGCTTTGCCCT	CGGAGCTGGTACAGAACTGG	Jacobs et al.^38^
Defensin-1	CTCTTGAGGAAGCGGCAACA	CAAGCGGCGTGATTGAGTTT	Jacobs et al.^38^
Tenecin-3	CATCACGACGGACATCTGGG	TAAATGTCCGCCTGGTTGGC	Jacobs et al.^38^
Tenmo-MIP	AAGGACTTGCACATCTGGGG	GTCGTATTGGGGTTCCAGCA	Lubawy et al.^37^

### Total haemocyte count

The total haemocyte count (THC) was determined as described previously by Lubawy et al.^40^. The samples of Tenebrio haemolymph (2 µL) were diluted in 20 µL physiological saline containing anticoagulant buffer (ACB: 4.5 mM citric acid and 9 mM sodium citrate) (PS:ACB, 5:1 v/v). Then, the prepared suspension was examined with a Bürker haemocytometer and a Nikon PrimoStar light microscope. To calculate the THC, 7–9 individuals were used in each of the treatments in three independent replications.

### Phagocytosis

A phagocytic assay was performed as described previously by Urbanski et al.^41^ and Lubawy et al.^40^. Phagocytosis was conducted in vitro using fluorescently labelled latex beads added to a suspension of haemolymph (Sigma‒Aldrich, Poznań, Poland). The haemolymph samples (2 µL) were added to a suspension of PS containing ACB (5:1 v/v) and latex beads, gently mixed and incubated for 30 min at room temperature on a microscopic slide coated with pol-L-lysine. After incubation, the samples were washed 3 times with PS and fixed with 4% paraformaldehyde solution for 10 min. After fixation, the samples were washed again and stained for 5 min with DAPI solution in PS (1:500) for nuclear visualization. Finally, the samples were washed and mounted using mounting medium (95% glycerol with 2.5% DABCO in PBS). The haemocytes prepared in this way were examined using a Nikon Eclipse TE 2000‐U fluorescence microscope (Nikon, Tokyo, Japan). The results are expressed as a percentage ratio of haemocytes with fully phagocytosed latex beads to the overall number of haemocytes visible on a single photo. In each biological repetition (8), the number of phagocytizing haemocytes was calculated from 5 random photos, giving 1261 to 1774 cells for each treatment. Overall, 9856 cells were counted.

### Phenoloxidase activity

Phenoloxidase (PO) activity was analysed based on the colorimetric method described by Sorrentino et al.^42^. Haemolymph samples (1 µL) were placed on Whatman No. 52 filter paper soaked with phosphate buffer (10 mM, pH 6.6) and DL-DOPA (2 mg/1 mL). After 30 min of incubation, the samples were air dried and scanned using Sharp AR 153EN (600 dpi, 8 bits, greyscale). The PO activity was determined by measuring the intensity of colour in the central part of the obtained spots. The results were expressed as the mean pixel value. To calculate PO activity, 10–12 individuals in 3 separate repetitions were used.

### Statistical analysis

For statistical analysis of the obtained data, we used GraphPad Prism software (Adam Mickiewicz University licence, version 9.0.0 for Windows, GraphPad Software, San Diego, California USA, www.graphpad.com). Before statistical analysis, the normality of distribution (the Shapiro‒Wilk test) was checked; if it passed, the parametric tests were used and if not, nonparametric tests were calculated. All data are presented as the mean values ± SEMs of the number of replicates (n) indicated. The statistical significance of differences between values of control insects and those exposed to experimental procedures were determined using a one-way ANOVA with Tukey’s post hoc, Student’s *t test* or Mann‒Whitney test. The survival curves were plotted using the Kaplan‒Meier estimator method, and the differences between the curves were estimated based on the log-rank (Mantel‒Cox) test. Differences were considered statistically significant if *p* ≤ 0.05 (*), *p* ≤ 0.01 (**), or *p* ≤ 0.001 (***).

## Results

### MIP expression level after cold

**Figure 2:**
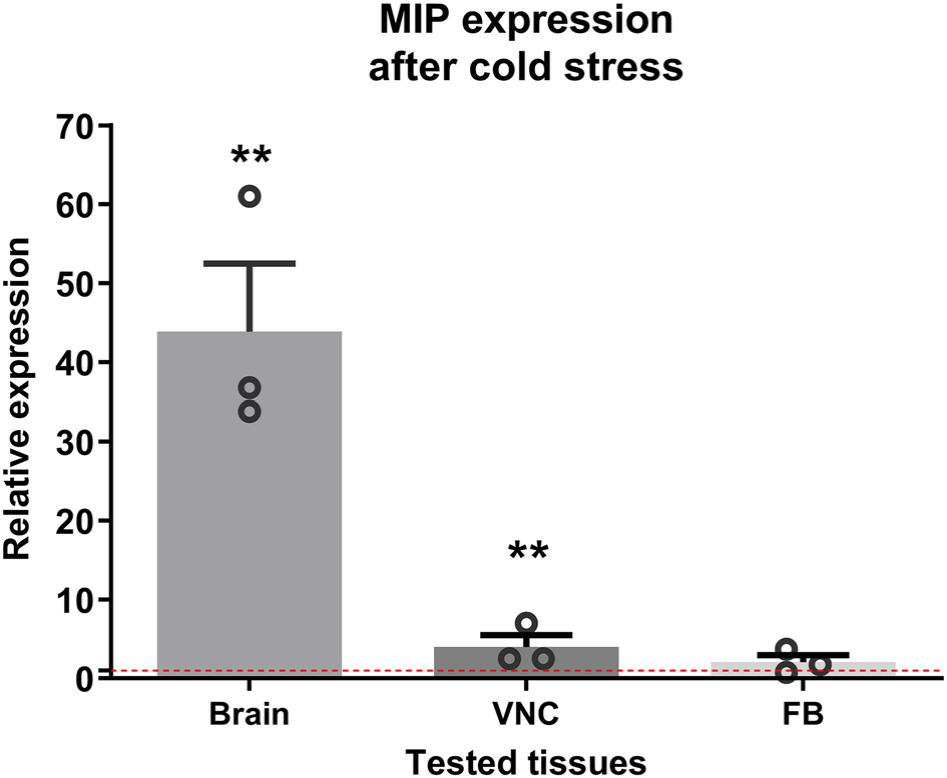
Expression level of *Tenmo-MIP* after cold stress in isolated brains, ventral nerve cords (VNC) and fat body (FB) of *Tenebrio molitor* L. Values are presented as the means ± SEM (n = 3 biological × 2 technical replications); **p ≤ 0.01 (Student’s *t test*), red line—expression level in control.

Cold stress treatment changes the expression of *MIP* in *T. molitor* tissues. The transcript level of the gene encoding this neuropeptide after one hour of stress increased 43.88-fold (± 6.12, *p* ≤ 0.01) in the brain and fourfold (± 1.78, *p* ≤ 0.01) in the VNC. Although the expression of the gene encoding Tenmo-MIP in FB also increased 2.08-fold (± 0.74), it was not statistically significant (Fig. 2).

### Chill coma recovery time

**Figure 3:**
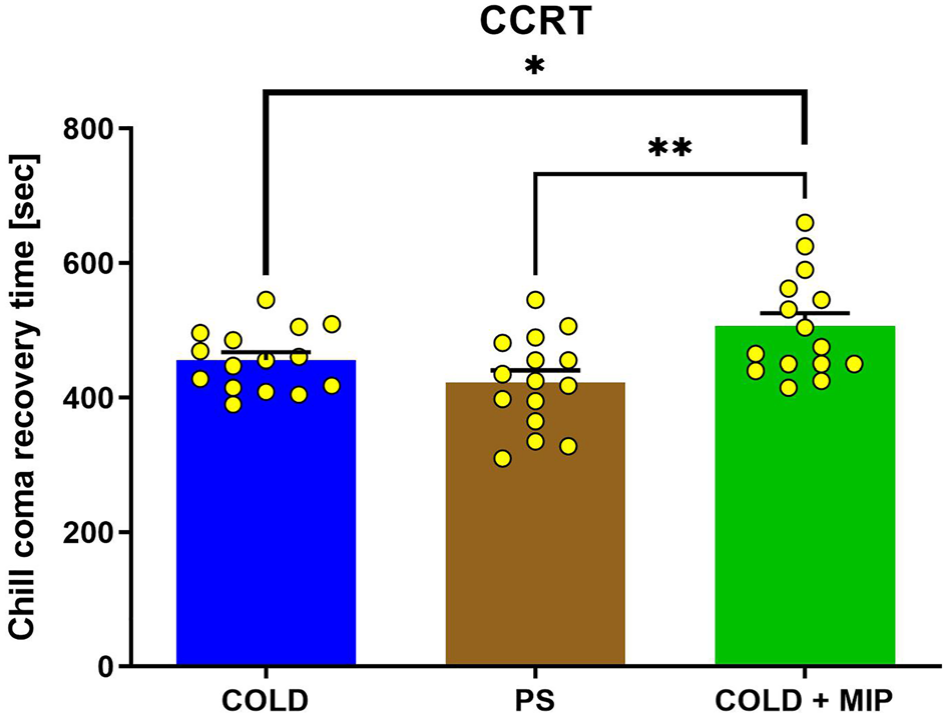
Chill coma recovery time (CCRT) of *T. molitor* after cold stress (COLD), injection of neuropeptide and cold stress (COLD + MIP) and injection of physiological saline (PS). Values are given as the mean ± SEM (n = 15), and yellow dots represent individual measurements. *p ≤ 0.05, **p ≤ 0.01 (Student’s *t test*).

The CCRT of beetles from the COLD treatment was 455.7 s. (± 11.73 s.), whereas those from PS and COLD + MIP were 422.7 s. (± 17.73 s.) and 505.8 s. (± 19.7 s.), respectively (Fig. 3). The CCRT in the PS group did not differ significantly from the COLD (*p* = 0.1311); however, the CCRT of the beetles from the COLD + MIP treatment was significantly longer than that from both the COLD and PS treatments (*p* = 0.0375 and *p* = 0.0040, respectively).

### Survival

**Figure 4:**
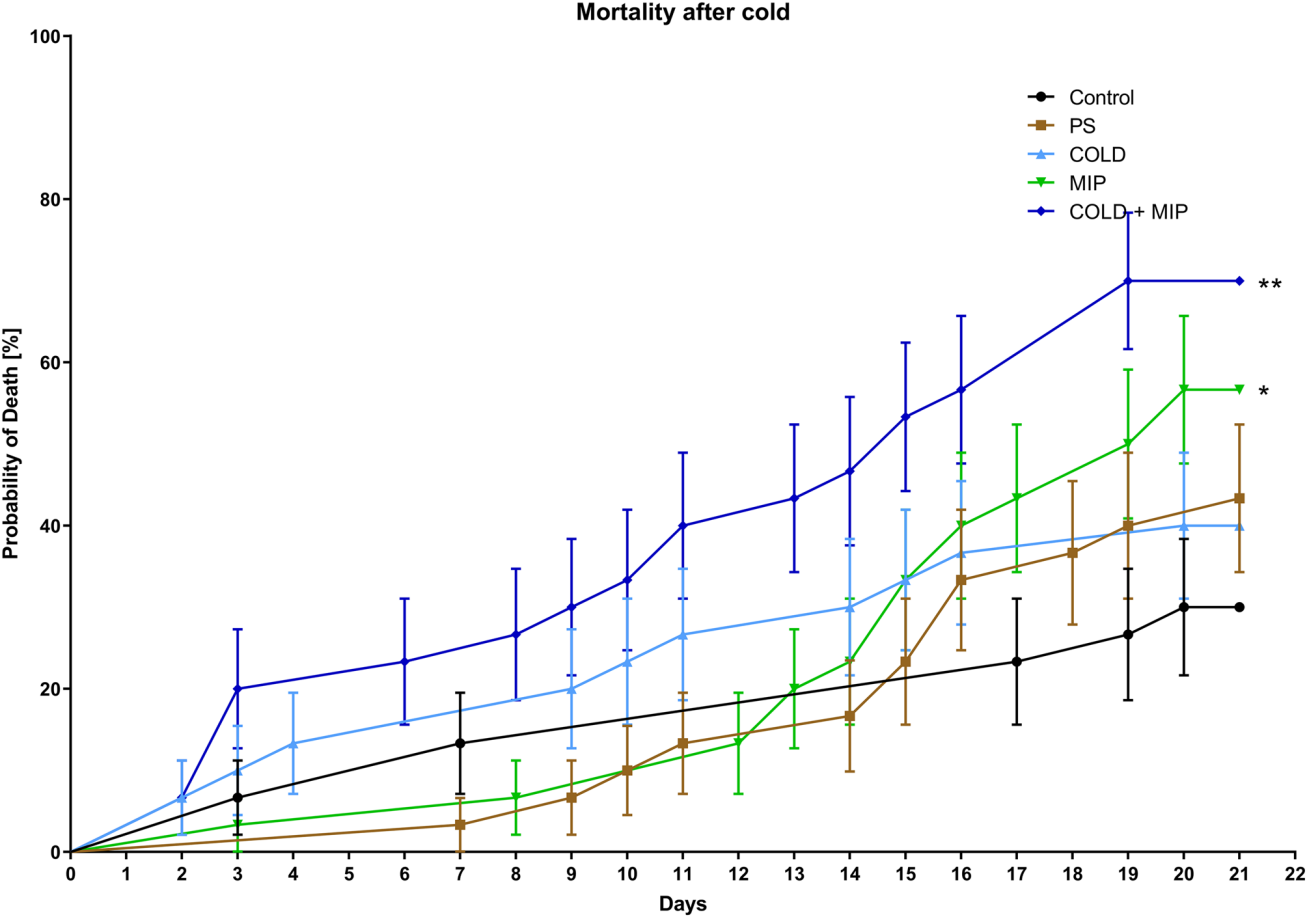
Mortality curves of *T. molitor* after cold stress (COLD, light blue line), injection of Tenmo-MIP 5 neuropeptide at a concentration of 10^–6^ M, giving a final concentration in the haemolymph of 10^–7^ M (MIP, green line), injection of neuropeptide and cold stress (COLD + MIP, dark blue line), injection of physiological saline (PS, brown line) and control (black line). Values are given as the mean ± SEM (n = 3, Note that because not all animals survived all the way through this experiment, some experimental groups only have one survivor after 21 days). The differences between survival curves were estimated based on the log-rank (Mantel‒Cox) test, *p ≤ 0.05; **p ≤ 0.01.

The analysis of the survival ratio showed no significant differences between PS injection and the control or COLD treatment and the control (Fig. 4). However, the injection of Tenmo-MIP 5 caused increased mortality in both the MIP and COLD + MIP treatments. The mortality increased by 26% (*p* = 0.0427) and 40% (*p* = 0.0012) compared to the control, respectively (Fig. 4). Increased mortality in the COLD + MIP treatment was also observed when this group was compared to PS (*p* = 0.0161).

### Expression level of immune-related genes

**Figure 5:**
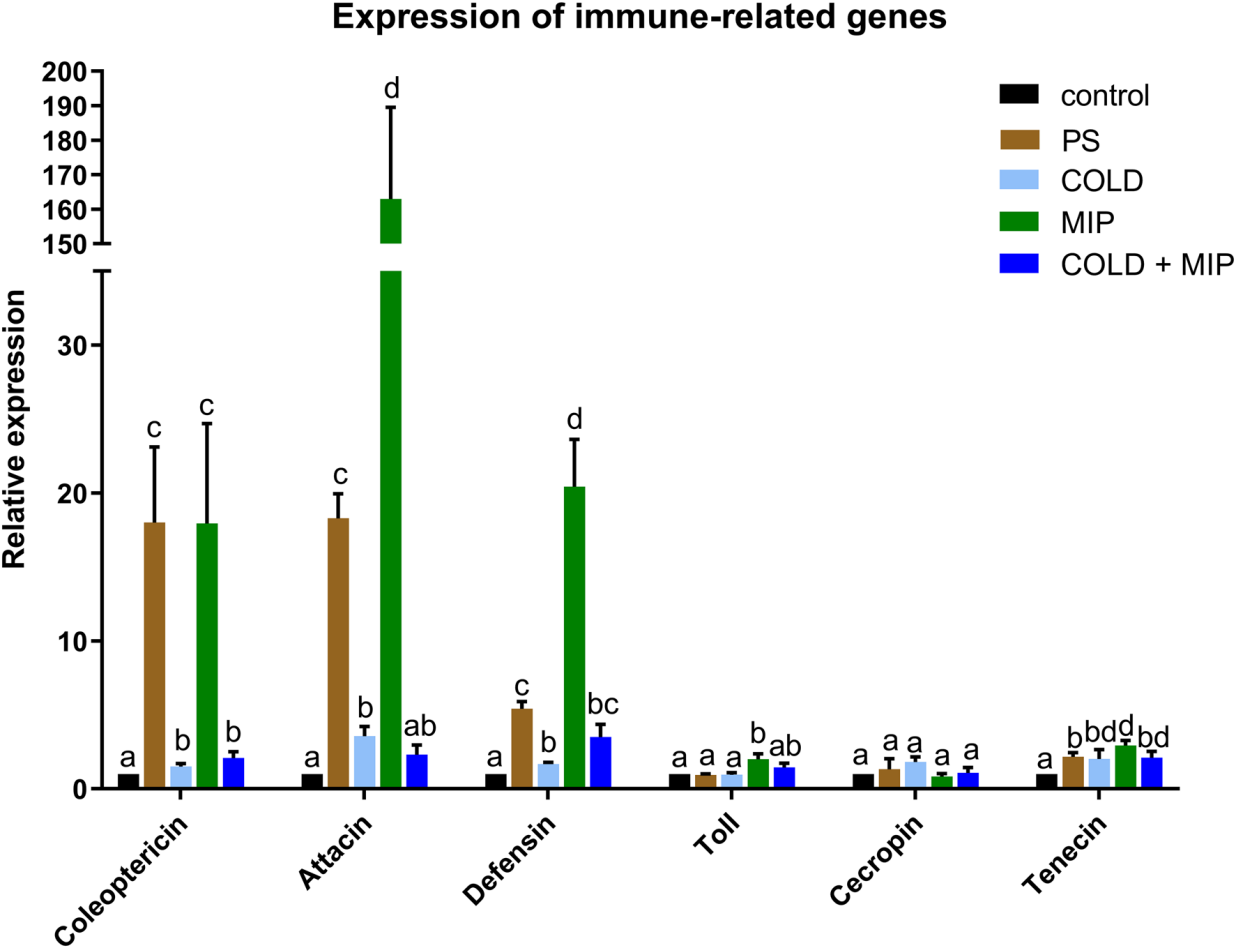
Quantitative analysis of the expression levels of immune-related genes in the fat body of *T. molitor* after cold stress (COLD), injection of Tenmo-MIP 5 at a concentration of 10^–6^ M, giving a final concentration in the haemolymph of 10^–7^ M (MIP), combined cold stress and neuropeptide injection (COLD + MIP) and physiological saline injection (PS). Values are given as the mean ± SEM (n = 3 biological × 2 technical replications). Different letters show statistically significant differences among treatments within each group at *p* ≤ 0.05 (one-way ANOVA).

Because insect FB produces and secretes antimicrobial peptides as well as additional components of the humoral immune response^43^, we measured the expression levels of six immune-related genes after each treatment, namely, Coleoptericin A, Attacin A, Defensin, Tenecin, Cecropin (antimicrobial peptides, AMPs) and Toll, and the observed changes in the tested genes are presented in Fig. 5. The COLD treatment caused a significant increase in the expression of four out of the tested genes, Coleoptericin, Attacin, Defensin and Tenecin, by 1.50-fold (± 0.21), 3.56-fold (± 0.66), 1.66-fold (± 0.14) and 2.03-fold (± 0.62), respectively (*p* = 0.0408, *p* = 0.0031, *p* = 0.0008 and *p* = 0.0469, respectively). The injection of neuropeptide (MIP variant) caused upregulation of most of the genes tested. The expression of Coleoptericin increased 17.95-fold (± 6.76, *p* = 0.0313), Attacin increased 162.98-fold (± 26.62, *p* = 0.0001) and Defensin increased 20.43-fold (± 3.20, *p* = 0.0001). The increase in Toll and Tenecin was also statistically significant; however, it was not as pronounced as the abovementioned genes. The upregulation was twofold (± 0.38, *p* = 0.0252) and 2.93-fold (± 0.33, *p* = 0.0313), respectively. In the case of combined treatments (COLD + MIP), an increase was observed for Coleoptericin, Defensin and Tenecin by 2.08-fold (± 0.44), 3.5-fold (± 0.86) and 2.1-fold (± 0.43), respectively (*p* = 0.0338, *p* = 0.0156 and *p* = 0.0156). Although injection of PS caused a significant increase in expression in the case of Coleoptericin (18.00-fold ± 5.11; *p* = 0.005), Attacin (18.28-fold ± 5.11; *p* < 0.0001), Defensin (5.42-fold ± 0.49; *p* = 0.005) and Tenecin (2.17-fold ± 0.29; *p* = 0.0027) relative to the control, the increase after peptide injection compared to physiological saline (MIP vs. PS) was significant for Attacin (*p* = 0.0003), Defensin (*p* = 0.0009), Toll (*p* = 0.021) and Tenecin (*p* = 0.0129) (Fig. 5), indicating that an increase after the peptide injection is not due to the injection procedure itself but due to the activity of the peptide.

### Phagocytosis

**Figure 6:**
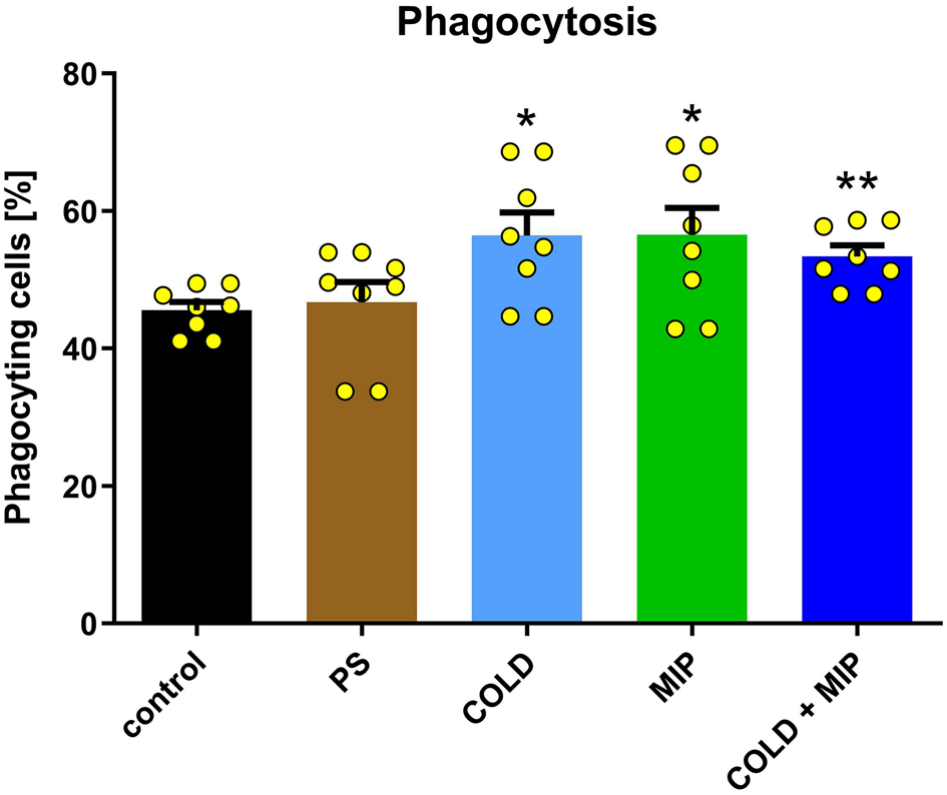
Phagocytosis activity of haemocytes in *T. molitor* after cold stress (COLD), Tenmo-MIP 5 injection at a concentration of 10^–6^ M giving a final concentration in the haemolymph of 10^–7^ M (MIP) and combined cold stress and neuropeptide injection (COLD + MIP) compared to control and injected with physiological saline (PS). Values are given as the mean ± SEM (n = 8). Yellow dots represent individual measurements. *p ≤ 0.05, **p ≤ 0.01 (Mann‒Whitney test).

The activity of *T. molitor* haemocytes to phagocyte changes significantly in all three variants (Fig. 6). Cold stress (COLD) and neuropeptide injection (MIP) similarly enhanced phagocytosis activity, increasing it by 10.66% and 10.99%, respectively (*p* = 0.0141 and *p* = 0.0260) compared to the control. When temperature stress was combined with peptide injection (COLD + MIP), phagocytosis activity also increased compared to that of the control, but only by 7.76% (*p* = 0.0038).

### Total haemocyte count

**Figure 7:**
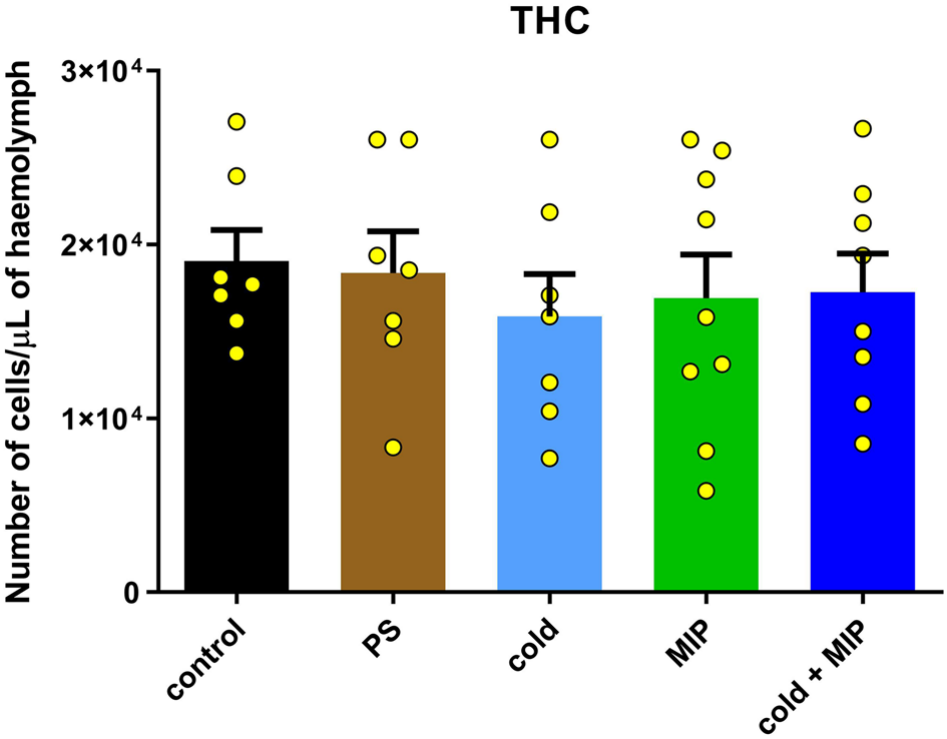
Total haemocyte count (THC) in *Tenebrio molitor* haemolymph after cold stress (COLD), Tenmo-MIP 5 injection at a concentration of 10^–6^ M giving a final concentration in the haemolymph of 10^–7^ M (MIP) and combined cold stress and neuropeptide injection (COLD + MIP) compared to control and injected with physiological saline (PS). Values are given as the mean ± SEM (n = 7–9). Yellow dots represent individual measurements.

We observed no significant changes in THC in all experimental variants (Fig. 7); however, a slight decrease in the number of cells was observed in the COLD, MIP and COLD + MIP groups.

### PO activity

**Figure 8:**
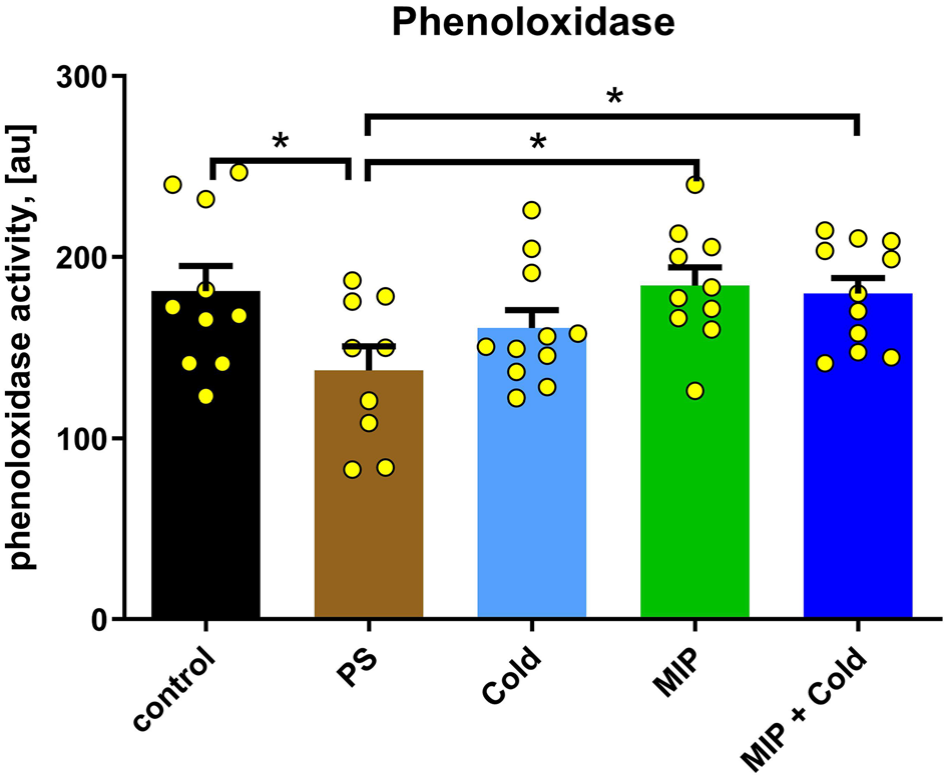
Phenoloxidase activity in *T. molitor* haemolymph after cold stress (COLD), Tenmo-MIP 5 injection at concentration of 10^–6^ M giving final concentration in the haemolymph of 10^–7^ M (MIP) and combined: cold stress and neuropeptide injection (COLD + MIP) compared to control and injected with physiological saline (PS). Values are given as the mean ± SEM (n = 10). Yellow dots represent individual measurements. *p ≤ 0.05 (Student’s *t test*).

In the case of phenoloxidase (PO) activity, an injection procedure of PS decreased the activity of this enzyme in *T. molitor* haemolymph (Fig. 8). Hence, we are comparing control individuals with those after cold stress treatment (Control vs. COLD) because the injection procedure was not performed in both of these groups, and we are comparing the individuals injected with neuropeptide (MIP) and combination of cold stress and peptide injection (COLD + MIP) with PS (MIP vs. PS, COLD + MIP vs. PS) because the injection procedure was performed in these groups. The COLD treatment caused a slight decrease in PO activity compared to the control; however, the difference was not statistically significant. After injection of Tenmo-MIP 5 at a 10^–6^ M concentration (MIP), the PO activity increased compared to PS by 34.15% (*p* = 0.0113); similarly, the activity of this enzyme increased after combined treatment (COLD + MIP) by 30.83% (*p* = 0.0131) when compared to PS (Fig. 8).

## Discussion

We explored the effects of the insect neuropeptide Tenmo-MIP 5 and cold stress on the immune system activity of the *Tenebrio molitor* beetle. We also investigated the crosstalk between the two in regard to immune response activation. Knowledge of the effect and role of insect neuropeptides on the immune response during cold stress is scarce, and to our knowledge, this is the first time that allatostatins have been explored. Allatostatins are considered homologues of vertebrate somatostatin, which are involved in the regulation of the immune system^13,32^. For this reason, the question arises whether MIP can affect the *T. molitor* immune system and whether it affects cold tolerance by cross-talk/cross-tolerance^21^. The results of the qPCR analysis support this supposition, as the transcript for the MIP precursor is strongly upregulated in the brain, the VNC and even in the fat body after cold stress.

Antimicrobial peptides (AMPs) are important effector compounds of innate immunity that are produced against pathogens. The mechanism of their action, which are usually nonspecific membrane interactions, blocks the buildup of long-term resistance of bacteria, viruses, fungi or parasites^44,45^. Significant changes in the expression of the genes encoding AMPs were observed in all experimental conditions, suggesting that both cold and peptide injection affect this type of humoral response. Such results were expected, as a number of studies show that stressful environments induce upregulation of immune-related genes^46,47^. Activation of AMPs expression after acute cold was observed previously in *D. melanogaster*^48^. Interestingly, the expression of most of the AMPs (Attacin, Defensin, Tenecin) also increased after injection of the neuropeptide Tenmo-MIP 5 and was much higher than that in cold-stressed insects. It must be mentioned that the injection of PS also has an effect on the expression of AMPs, showing in some cases a higher effect than cold itself. However, even when taking into consideration the increase in AMPs expression after the injection procedure only (PS group), the effect of the peptide on their expression was still observable (except for Coleoptericin, Fig. 5). MIPs are capable of inhibiting juvenile hormone (JH) synthesis in tenebrionid beetles^49^. JH in turn is involved in the regulation of insect immunity, with an emphasis on AMPs expression^50^. In *D. melanogaster,* JH exerts a suppressive effect on the expression of AMPs both in vitro and in vivo^51^. As MIPs inhibit the production and release of JH in the beetle retrocerebral complex^52^, which in turn is responsible for maintaining an appropriate level of insulin-like peptides (ILPs)^53^, it may also explain the upregulation observed in this study. Additionally, allatostatins regulate gut activity in a number of ways^54,55^, and disturbance of normal gut activity, e.g., transcription factors expressed in the gut, leads to changes in AMPs expression^56,57^. However, when combining cold stress and peptide injection, the results also show AMPs upregulation, yet not as profound as in the case of peptide injection alone. Although MIP seems to be a very potent AMPs activator, the trade-offs experienced by the immune system during acute stress^48^ seem to exceed the activity of the peptide.

Haemocytes are primarily responsible for an insect’s immune response. Among others, they are responsible for phagocytosis, encapsulation or release of PO^58^. Studies have shown that cold stress significantly affects cellular responses. In the beetle *Nicrophorus vespilloides,* Urbanski et al.^59^ showed that cold gradually decreases the number of haemocytes during winter, as it also occurs in overwintering *Pyrrharctia isabella*^60^. On the other hand, in *Eurosta solidaginis* and *Curculio* sp. cold does not affect the number of haemocytes^60^. Similarly, in *T. molitor,* acute stress did not cause changes in haemocyte concentrations (Fig. 7). The wide variation in responses indicates the presence of species- or condition-specific factors that moderate the haemocyte response to cold stress. Peptide injection together with cold stress also did not change the THC. However, again by modulation of JH, MIPs may affect haemocytes in a different manner, e.g., their morphology or adhesive abilities and not only their number. Hepat and Kim^61^ showed that JH influences the process of haemocyte spreading and nodulation in the *T. castaneum* beetle.

The effect of Tenmo-MIP 5 on phenoloxidase activity may be a result of the influence of MIP neuropeptides on the functioning of the retrocerebral complex, which secretes, e.g., AKH—neurohormone stimulating the activity of PO^62,63^. Neurotropic activity and the ability to regulate the secretion of neuropeptides from the neurosecretory complex by allatostatins were demonstrated by Hentze et al.^64^ and Clark^65^. The stimulation of PO activity was observed in our studies both when only MIP was administered and when injection was combined with cold (Fig. 8). It was shown in other species that higher PO activity during cold is most likely needed for better survival of adverse conditions, as was shown in *Aquarius najas*, in which an enhanced encapsulation (mediated by PO) response is associated with higher winter survival^66^. The higher levels of PO may be the result of a higher concentration of proteins in the haemolymph, as was shown for overwintering queens of *Bombus lucorum*^67^. However, for this process to occur, acclimatization is most likely necessary, as indicated by Urbanski et al.^59^ in another beetle. This may explain the lack of increased PO activity in our study in the COLD variant. The activation of PO after peptide injection seems to be expected, as the activity of this enzyme seems to be regulated by neuropeptides^39,62,63^. Mavrouli et al.^68^ also showed that prophenoloxidase (proPO) is involved in phagocytosis. Phagocytosis is an evolutionarily conserved process that in insects is mediated by haemocytes. Foreign particles, such as microorganisms/latex beads, are recognized by pattern recognition receptors (PRRs), both soluble in the haemolymph (working as opsonins) and fixed on the surface of haemocytes, and are internalized into a phagosome that transforms into a phagolysosome, where they are digested. The results of our experiments revealed that Tenmo-MIP 5 and cold stress similarly enhanced the process of phagocytosis in *T. molitor* (Fig. 6). The enhancement of phagocytosis during cold was also observed in *N. vespilioides*, in which, as suggested by the authors of that study, higher phagocytosis was associated with a higher level of PO^59^.

**Figure 9:**
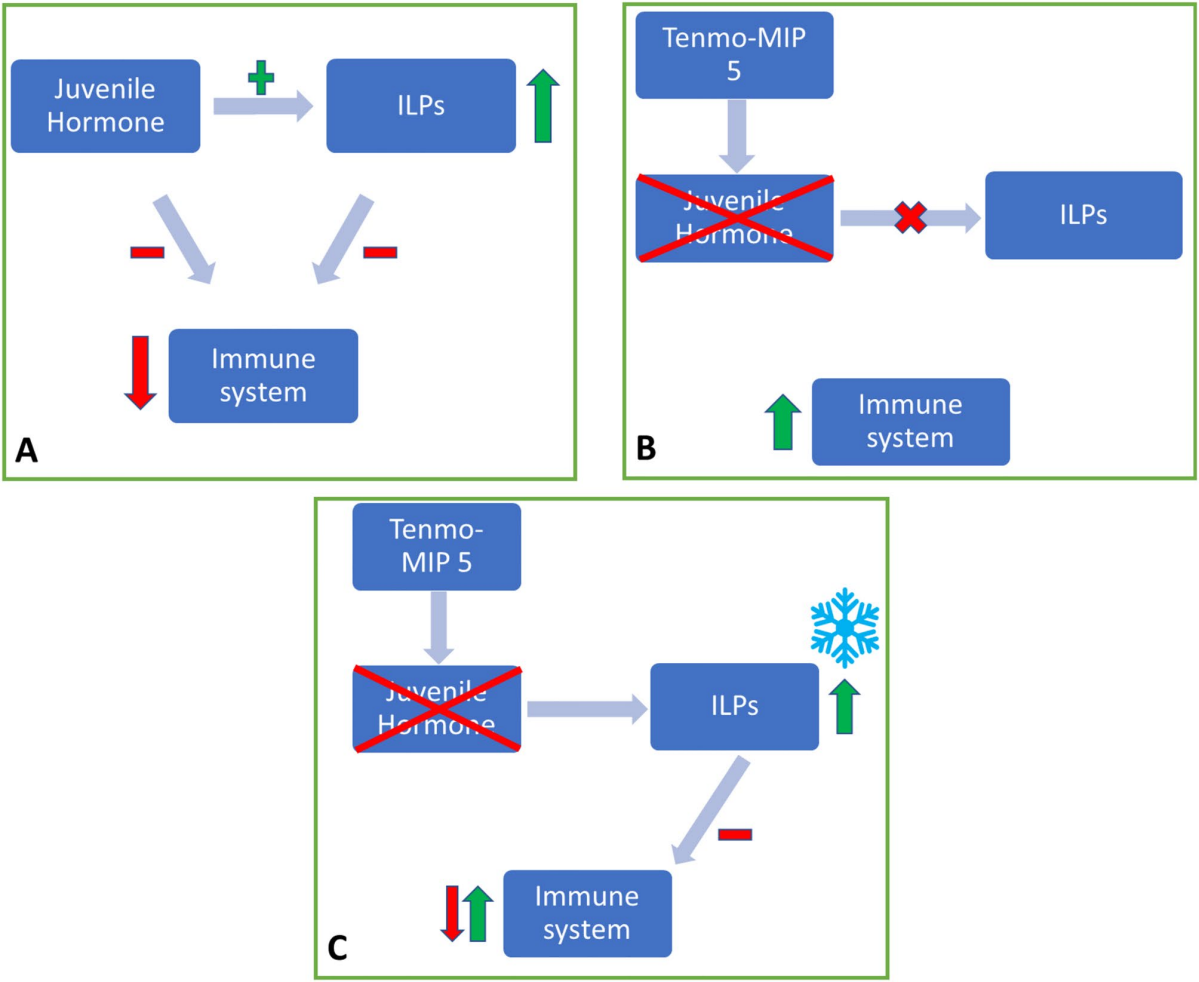
Scheme representing the possible effect of the Tenmo-MIP 5 injection on immune system activity, taking into account the JH/ILP feedback loop. Before peptide injection, JH and ILPs suppress the activity of the immune system (**A**). The injection of Tenmo-MIP 5 causes an inhibition in the production and release of JH from the retrocerebral complex of *T. molitor,* thereby reducing the effect of this hormone on the level of ILPs. This leads to inhibition of the suppressive role on the immune system, causing its higher activation (**B**). Cold stress, being a positive regulator of ILPs, causes an increase in the level of ILPs, resulting in partial suppression of immune system activity (**C**), which may explain the mitigation of the Tenmo-MIP 5 injection by cold stress of almost all tested parameters observed in our study.

Cold stress did not significantly affect the mortality of the insects, whereas injection of the peptide and combination of peptide injection and cold stress did significantly affect mortality (Fig. 4). Additionally, the time to recover from chill coma measured in our study is very similar to CCRT values obtained by other researchers in *T. molitor*^69^. Insects injected with Tenmo-MIP 5 took longer to recover from chill coma, while the group injected with PS showed no differences compared to the control (Fig. 3). This suggests that MIP lowers the cold tolerance of *T. molitor* and that the observed effect is not due to the injection itself. This might be due to several reasons. It is well established that a central requirement for chill tolerance is the maintenance of ion homeostasis, which is achieved through osmoregulatory mechanisms. In insects, osmoregulation is maintained via the Malpighian tubules (MTs) and the gut^7,8,70^, which interplay. From other studies, it can be concluded that neuroendocrine factors play critical roles in osmoregulation and therefore cold tolerance. For example, members of the CAPA family modulate cold tolerance via effects on MTs^71,72^, and they can also modulate the immune response^73^. When injected, CAPA has antidiuretic activity in *D. melanogaster,* reducing tubule K^+^ clearance rates and chill tolerance by significantly increasing CCRT^74^. Chintapalli et al.^75^ proposed the neuropeptide control mechanism for the tubules by MIPs because allatostatin B-reactive cells are found at the junction between the anterior and middle midgut^54,55^. The injection of peptide may disturb the interplay between MTs and the gut, leading to lower cold tolerance (expressed by CCRT) and to increased mortality. Another possibility is the disturbance of the JH titter by allatostatins. As mentioned before, JH synthesis and release are regulated by MIPs in Tenebrionidae beetles^52^. Studies suggest the presence of a feedback loop between JH and the insulin/IGF signalling system (IIS)^76^. IIS also play a role in the response to a variety of stresses, such as starvation or oxidative stress with temperature stress^10,77^. In turn, JH serves as a positive regulator of IIS, whereas IIS negatively regulates JH levels^10,77^. IIS by insulin-like peptides (ILPs) exerts direct activity on the immune system of insects, affecting all of the main immune-related pathways (Toll, Imd, JAK/STAT) (Fig. 9), causing, for example, changes in AMPs synthesis or modulating the cellular response^53^, playing a role in the immediate response as well as in the long-term fitness consequences for the individual^50^. Overall, both of the abovementioned hormones are considered to be negative regulators of the immune response^50^. Disturbing the MT-gut interplay and/or JH-IIS feedback loop by allatostatins may cause increased mortality during cold stress.

## Summary and conclusions

In summary, in the present study, the effect of cold stress and MIP on the immune response of *T. molitor* was tested. The obtained results show that both cold stress and peptide injection, separately or together, affect the cellular and humoral response in the insect body. However, most of our experiments demonstrate that cold mitigates the effect of peptide injection when combined (COLD + MIP). We believe that the observed results may be due to the effect of Tenmo-MIP 5 on the JH-IIS feedback loop affecting JH levels^52^ and/or insulin signalling^78^, which we tried to depict in Fig. 9. Both JH and ILPs are considered immune system suppressors. Tenmo-MIP 5 causes the inhibition of synthesis and release of JH from the retrocerebral complex in the insect brain^52^, which in turn is a positive regulator of ILPs and is responsible for maintaining an appropriate level of these neuropeptides^53^. This leads to inhibition of the suppressive role on the immune system, causing its activation (observed in most tested parameters after peptide injection). However, cold stress is also a positive regulator of ILPs, increasing their levels in insect haemolymph^79^. When insects are exposed to cold stress after peptide injection, there is a high probability that, despite JH inhibition by Tenmo-MIP 5, insulin-producing cells are activated (Fig. 9). This may explain the mitigation of the Tenmo-MIP 5 injection by cold stress of almost all tested parameters observed in our study. Second, MIPs, by affecting the functioning of the gut, where they are expressed, can disturb the MT-gut interplay needed for proper cold resistance and immune activity. However, cold stress tolerance and immune activity are complex, linked processes regulated by a number of feedback loops, and the role of MIPs can be multiple in the regulation of these finely tuned processes. Hence, more studies on this topic are needed, and our research can be considered the beginning of further work related to the neurohormonal regulation of these two processes.

## Data Availability

The datasets used during the current study are available from the corresponding author on reasonable request.
